# The control of hyperglycemia by a novel trypsin resistant oral insulin preparation in alloxan induced type I diabetic mice

**DOI:** 10.1038/srep26789

**Published:** 2016-05-26

**Authors:** Sarbashri Bank, Arjun Ghosh, Suman Bhattacharya, Smarajit Maiti, Gausal A. Khan, Asru K Sinha

**Affiliations:** 1 Sinha Institute of Medical Science & Technology, 288-Kendua main road, Baishnabghata, Garia, Kolkata-700084, India; 2 Cell & Molecular Therapeutic Lab, Dept. of Biochemistry, Vidyasagar University, Midnapur-721102, India; 3 Defence Institute of Physiology and Allied Sciences, Lucknow Road, Timarpur, Delhi-54, India

## Abstract

A trypsin resistant oral insulin preparation was made by incubating insulin for 2 h at 23 °C with previously boiled cow milk at 100 °C that was coagulated with 0.6 M acetic acid. The precipitate was resuspended in the same volume of milk. The immunoblot analysis of the suspended proteins treated with 200 ng of trypsin/ml for 3 h demonstrated that the 80.1% of the insulin in the suspension survived the proteolytic degradation compared to 0% of the hormone survived in the control. The feeding of 0.4 ml (0.08 unit of insulin) of the resuspended proteins followed by 0.2 ml of the same protein to alloxan induced diabetic mice maximally decreased the blood glucose level from 508 ± 10 mg/dl to 130 ± 10 mg/dl in 7 h with simultaneous increase of the basal plasma concentration of insulin from 3 ± 1.1 μunits/ml to 18 ± 1.5 μunits/ml. In control experiment the absence of insulin in the identical milk suspension produced no hypoglycemic effect suggesting milk was not responsible for the hypoglycemic effect of milk-insulin complex. Coming out of insulin-casein complex from the intestinal gut to the circulation was spontaneous and facilitated diffusion transportation which was found from Gibbs free energy reaction.

Insulin, a hypoglycemic protein hormone discovered by Best and Banting^1^ has an essential role in the carbohydrate metabolism in the transduction of energy for the survival of all forms of animal lives^2^; indeed there is no known alternative to insulin that can replace the protein hormone. According to IDF DIABETES ATLAS, (Sixth edition), the majority of the 382 million people with diabetes are aged between 40 and 59, and 80% of them live in low- and middle-income countries. All types of diabetes are on the increase, type 2 diabetes in particular: the number of people with diabetes will increase by 55% by 2035.

Despite the life saving properties of insulin, when there is a systematic deficiency either due to the systemic impaired insulin synthesis (type 1 diabetes mellitus) or the system itself became resistant to the hormonal effect (type 2 diabetes mellitus) the hormone must be injected in the system from external sources as insulin taken by mouth is rapidly destroyed in the digestive tract particularly due to tryptic digestion, and, as such the needed hormone is not adequately available in sufficient amount to make up for the systemic deficiency of the hypoglycemic protein. Although various efforts have been made to prepare orally active insulin for the control of hyperglycemia^3^, the availability of oral insulin preparation has not yet been achieved.

We report herein an orally active trypsin resistant insulin preparation by using commercially available any kind of recombinant insulin by using a simple household procedure that can be carried out anywhere by using minimal facilities without using high technology or expensive instruments that requires trained personnel with expertise in protein chemistry.

## Methods

### Chemicals

Insulin was prepared and purified by chromatographic method^4^ and the same effect was also found from insulin (Biphasic isophane insulin injection I.P.Human Mixtard) with different trade names from market, casein, trypsin, HRP-conjugated secondary antibody and primary insulin antibody were from Sigma-Aldrich, ELISA maxisorb plate from Nunc-roskilled. All the other chemicals were of analytical grade.

### Ethical clearance

Ethical clearance for this experiment “**The control of hyperglycemia by a novel trypsin resistant oral insulin preparation in alloxan induced type I diabetic mice”** and all the experimental protocol were approved by INSTITUTIONAL REVIEW BOARD, HUMAN AND ANIMAL RESEARCH ETHICS COMMITTEE, Sinha Institute of Medical Science & Technology, Kolkata, India. Twenty mice were used for this purpose under the strict supervision of a licensed veterinarian. No deviation was permitted from the protocol. All the experiments were performed according to Helsinki declaration, 1964.

### Preparation of oral insulin

1 mL of cow’s milk was boiled (100 °C) for 2 minutes and cooled to room temperature. Then 100 μlit of insulin (0.08 unit of insulin) was added to the cooled milk and incubated for two and half hours at room temperature (23 °C). After incubation, milk-insulin mixture was precipitated by 10 μL (0.6 M) acetic acid or by lemon juice and let it stand in that condition for 45 min. After precipitation, supernatant can be carefully decanted and added with same amount of cooled and boiled milk to the milk-insulin precipitated mixture to make up the volume 1ml (final).

### Experimental animal

White albino Swiss adult mice (*Mus musculus*) (M-10, F-10) aged (3–4 months) wt. 25–30 gm were used in the study. A licensed veterinarian checked them to see that they were free of the disease.

### Preparation of alloxan induced diabetic mice

Alloxan (Sigma Aldrich) was injected (150 mg/kg) to the fasting mice. Then the mice were kept with sufficient food so that they could not hypoglycemic. After 72 h, experiments were done by the diabetic mice.

### Determination of the distribution of insulin in orally fed milk-insulin from different parts of the diabetic mice

The milk-insulin preparation (0.4 mL) as described above was fed to the alloxan induced diabetic mice. After 3 h and 7 h blood sample (0.1 mL) were collected from tail vein, left and right superior vena cava, hepatic vein, and femoral vein. At the same time blood sample was also taken from appropriate control diabetic mice where only insulin (i.e. not incubated with milk) was fed to the mice. To minimize the pain and discomfort of the mice, morphine was administered to the mice. The amounts of insulin was quantitated in each case by ELISA.

### Preparations of intestinal loops filled up with milk-insulin preparation solution

Normal adult white mice (3–4 months) were killed by cervical dislocation and the small intestine was cut out. The intestine was cut into same size (1–1.5 inch) in five pieces. The inner portion of the each pieces of intestine was washed to clean well by Tyrode’s buffer. One end of the piece of intestine was bound with silk cord and insulin-preparation solution bound to casein as described above was pipetted inside the loop then the other open side of the intestine was similarly bound tightly. These intestinal loops were next kept separately submerged in 2 mL of tyrod’s buffer solution with glucose and both the milieu and the intestinal loop were mildly shaken at 16 Hz oscillation at 37 °C and at 1, 2, 3, 4, 5, 6, 7 h intervals a small portion (0.2 mL) of the milieu was withdrawn and the insulin in the milieu was determined by ELISA by using insulin antibody as described above.

### Immunoblot analysis of milk-insulin mixture treated with trypsin

Presence of insulin in the trypsin treated milk-insulin mixture as described in the manuscript was analyzed by immunoblot using by insulin antibody^5^. The transfer buffer used in the experiment contains Tris, Glycine in 20% Methanol while TBST (TBS with 0.1% Tween-20) was used for washing. Incubation buffer was contained Tris and NaCl (TBS). The membrane was blocked with 5%BSA in TBS. Ponceau (1 gm/100 mL) was used to check the transfer of protein from gel to membrane.

### Scatchard plot analysis of equilibrium binding of insulin with casein

Pure casein in PBS (phosphate buffer saline) was incubated with different concentration of insulin for 150 minutes (for maximum insulin binding optimal incubation time). 500 μL incubation mixture was filtered over glass fiber membrane (GF/C, Sigma Aldrich) by using Millipore filtration as described before^6^. The casein with the bound insulin remained adhere to the filter of GF/C membrane and unbound insulin passed through the membrane under mild vacuum condition^7^,^8^. The membrane filter was washed with 3 vol of reaction buffer. Next, the bound insulin was eluted from the filter by washing in 1vol of reaction buffer containing 0.05% TritonX-100. Amount of insulin in the filtrate was determined by ELISA using insulin antibody.

Scatchard plot analysis of equilibrium binding of insulin was constructed and dissociation constant (K_d_) and number of insulin binding (n) to the casein was analyzed by using Microsoft Office Excel programme.

### Statistical analyses

Glucose level and insulin concentration were shown here in mean ± standard deviation (S.D) of at least 10 different experiments. The relation between the decrease of glucose level and increase of insulin concentration was measured by Pearson’s Correlation coefficient test determined by ‘r’ value. Scatchard plot analysis of the equilibrium binding of insulin was analyzed by using Graphpad prism software and Microsoft Office Excel were used to determine the dissociation constant (K_d_) and the number of binding sites (B_max_) in the casein molecule.

## Results

### When 400 μl and followed by after 3 h 200 μl of milk-insulin preparation solution was fed to the alloxan treated diabetic mice, the glucose level was found to decrease from 508 mg/dL to 130 mg/dL

**Figure 1:**
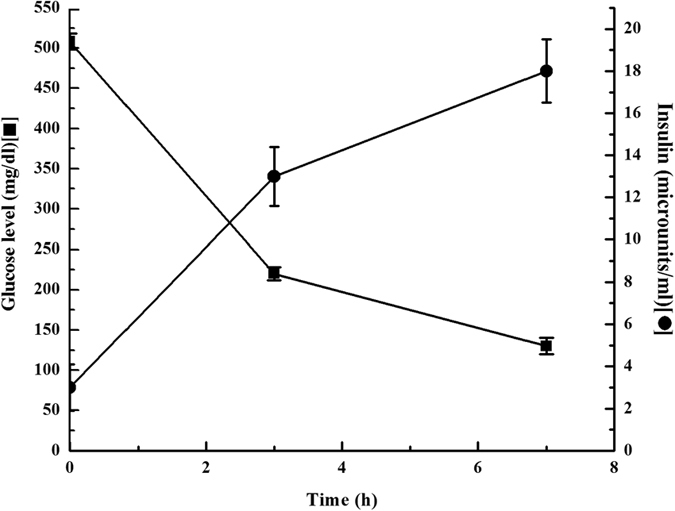
Level of glucose and insulin after oral ingestion of insulin preparation to the alloxan treated diabetic mice. After ingestion of 0.4 mL insulin preparation (containing 0.08 unit insulin) and followed by after 3 h, ingestion of 0.2 mL of insulin preparation to the alloxan treated diabetic mice, the glucose level was decreased from 508 ± 10 mg/dL to 220 ± 8 mg/dL after 3 hr and glucose level was decreased to 130 ± 10 mg/dL after 7 hr and insulin concentration were 3 ± 1.1 μU of insulin/ml before the feeding of the insulin preparation was found to increase to 13 ± 1.4 μU of insulin/ml at 3 h and to 18 ± 1.5 μU of insulin/mL at 7 h respectively.

When 400 μL of the insulin preparation (containing 0.08 U of insulin) in the milk as described before and followed by 3 h later 200 μL of the same preparation (0.04 U insulin) was fed to alloxan treated mice, that is reported to produce diabetic mice mimicking type I diabetes mellitus in human^9^. It was found that the plasma glucose level of these mice which was decreased from 508 ± 10 mg/dL (before the insulin preparation was fed) to 220 ± 8 mg/dL at 3 h and to 130 ± 10 mg/dL after 7 h (Fig. 1) (p < 0.001, n = 20). Thus the feeding of milk-insulin preparation to the alloxan treated diabetic mice was found to control the hyperglycemic effect for 11 h. It was further noticed that insulin preparation did not produce any discernable pathological problems in mice; no sickness was determined by veterinarians. Insulin preparation did not produce any toxic effect at least for a month. Insulin preparation keeps its activity at least for 7 days at 4 °C but at room temperature, it was found to active only 3–4 h.

In a separate experiment, using the same mice, when the plasma insulin concentration was determined at different hours after the feeding of the insulin preparation as described under Fig. 1, it was found that the plasma insulin concentration in the alloxan treated mice which was 3 ± 1.1 μUnit of insulin/ml before the feeding of the insulin preparation was found to increase to 13 ± 1.4 μUnit of insulin/ml at 3 h and to 18 ± 1.5 μU of insulin/ml at 7 h (Fig. 1), the Pearson test for the correlation (r) between the decrease of plasma glucose level and the increase of the plasma insulin concentration was determined to be r = −0.9946, (two tailed p value, p < 0.0001) indicating the increase of insulin concentration was highly but inversely correlated to the decrease of plasma glucose level. As the alloxan treated mice were incapable of synthesizing insulin in the system^9^, it was concluded that the increase of the plasma insulin concentration was due to the feeding of the milk insulin preparation from which the hypoglycemic hormone in digestive tract in these animals was able to enter into the circulation to produce the insulin effect in the control of hyperglycemia to normoglycemic level in the type I diabetic mice model. It was calculated that the almost 2.3% (in the 25 g mice) of the insulin that was present in the milk suspension would have entered into the circulation from the digestive tract in the animal.

### The distribution of insulin at different parts of the body after feeding the oral insulin (milk-insulin) to the diabetic mice

To determine whether the insulin was actually distributed in different parts of the body, the blood samples were taken from different parts of the body (as described in Table 1) at different time intervals and enzyme linked immunosorbant assay was performed by using insulin antibody. It was found that the insulin (μU/ml) was distributed in different parts of the body and increased with the time (Table 1). From the ELISA it was resulted that the demonstrated insulin was remained the more or less same.

### The direction of the movement of milk-insulin preparation solution was calculated from Gibbs free energy (∆G) equation

The direction of movement of the milk-insulin preparation was based on thermodynamics of the system. The diffusion of a substance between two sides of a membrane: A(out) ⇋ A(in), thermodynamically resembles a chemical equilibrium. Here, A = Casein-Insulin complex.

A difference in the concentrations of substance on two sides of the membrane generates chemical potential difference:


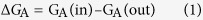


[∆G_A_ is the chemical potential of A = Casein-Insulin complex, expressed in partial molar free energy]

By putting the value of ∆G_A_ = RTln[A] in equation….(1)


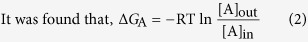


By putting the value of A = Casein-insulin complex in equation…(2)


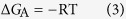


[as conc. of insulin in the external milieu [A]_out_ = 0, conc. of insulin in intestinal loop [A]_in_ = 12 × 10^4^ μU/ml]

By putting the value of Gas constant (R) and temperature (T) in Kelvin scale in equation…(3)

∆G_A_ = −8.314 J mol^−1^ K^−1^ × 310 K [ as, R = 8.314 J mol^−1^ K^−1^ and T = 37 °C = 310 K]

then, ∆G_A_ = −2577 J. mol^−1^

As ∆G_A_ <0, i.e negative value of ∆G indicated that the coming out of casein-insulin complex to the circulation (milieu here) was a spontaneous and energy independent process facilitated by diffusive transportation (natural entropy) (see Supplementary Fig. S1).

As ∆G_A_ <0, from the experiment, it was found that the rate of efflux of the insulin (K_1_) from the intestinal loop to the external milieu (Tyrod’s buffer) was 2.62 μUnits/ml/h where as on the other hand entering of insulin from the milieu in the intestinal loop was undectable (0 μUnits/ml/h) K_2_ = 0, because coming out of insulin from the intestinal loop was found to increase at different time and like a hyperbolic curve was constructed (Supplementary Fig. S1, indicates the process was not a simple diffusion but was facilitated diffusive transportation) i.e. no backward movement was found to occur in this case. It was inferred that casein-insulin complex that transported out from the intestinal wall was a spontaneous process.

The possibility whether insulin-casein complex itself was capable of synthesizing insulin in the intestinal wall was carried. In order to purify the insulin mRNA from external milieu, the mRNA from milieu was applied to the column of oligo(dT)-cellulose and optical density measured at 260 nm. The fractions were eluted by the buffer containing 10 mM Tris (pH 7.5); 1 mM EDTA and 0.05% SDS. The fractions were translated *in vitro* by using plant ribosomes, mixture of all 20 amino acids (1 μM each) and ATP (1 mM) as described^10^. The fractions showing the highest activity for the *in vitro* translation of insulin were pooled. Synthesis of cDNA was performed by RT-PCR (Reverse transcriptase-polymerase chain reaction) of the isolated mRNA of external milieu for the amplification of the synthesized product by using insulin gene specific primers, but no newly synthesized insulin was found in the intestines used in the experiment.

### Extended presence of insulin in the trypsin treated oral insulin preparation solution

**Figure 2:**
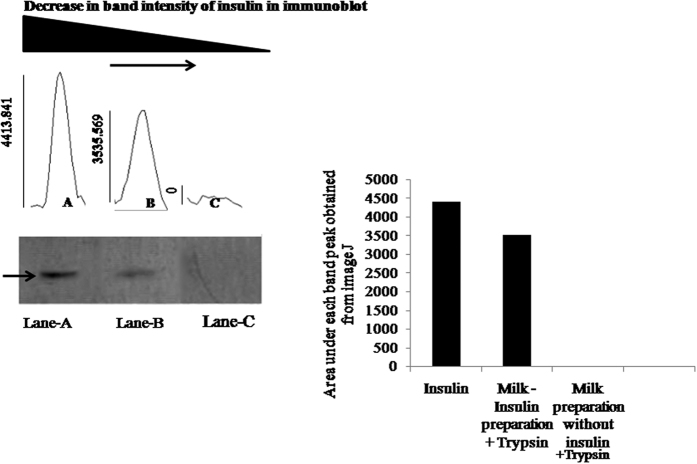
Western blot analysis of trypsin treated oral insulin preparation solution. This figure demonstrated the extension of insulin in trypsin treated insulin preparation solution. It was observed that trypsin was unable to degrade insulin in milk-insulin preparation solution. Here, Lane-A represented the pure insulin (insulin alone) band by western blot analysis where no trypsin was added and an arrow (→) indicated the positive band of insulin in Lane-A; Lane-B represented the insulin band in trypsin treated insulin preparation after 180 min and Lane-C represented trypsin treated milk preparation without insulin by western blot analysis using insulin antibody. From the Image-J analysis, it was found that 80.1% of insulin remained unaltered in lane-B compared to insulin (alone) in lane-A after 180 min where as no positive band was found in Lane-C.

The above results also suggested that the added recombinant insulin in the milk suspension survived the tryptic digestion in the digestive tract of the animals. To determine whether insulin in the milk suspension was indeed resistant to the proteolytic degradation catalysed by trypsin, the insulin preparation in the milk suspension was incubated with 200 ng/ml pure trypsin (physiologic level)^11^,^12^ for different time at 37 °C. After incubation, the extend of the proteolytic degradation induced by trypsin was determined by immunoblot analysis as described in the Method section. It was found that even after 3 h of incubation at 37 °C almost 80.1% of the insulin survived the tryptic digestion compared to the 0% insulin survived the trypsin effect (insulin alone) (Fig. 2).

### Scatchard plot of the equilibrium binding of insulin to the casein

**Figure 3:**
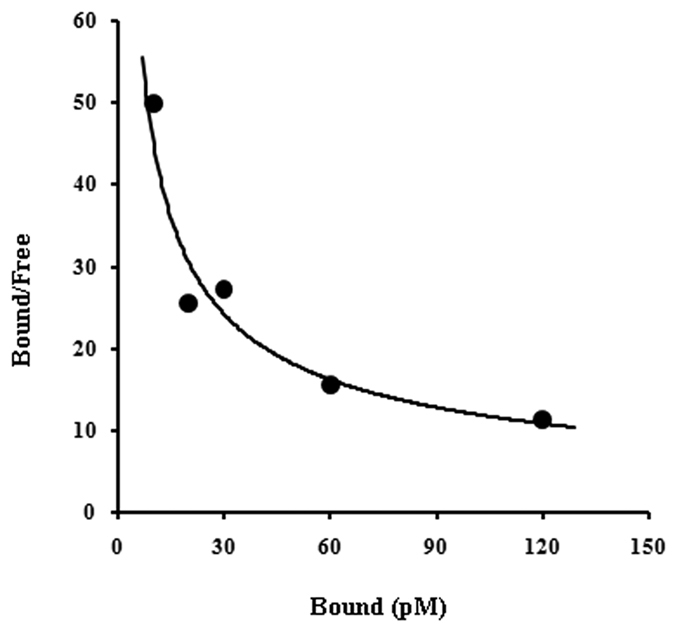
Scatchard plot analysis of equilibrium binding of insulin to the casein in presence of different concentration of insulin. Casein was incubated with different concentrations of insulin for 150 min. Unbound insulin was separated from casein bound insulin using by Millipore filtration unit as described in Methods and Materials. Bound insulin was released from casein in presence of 0.05% Triton-X-100 and the amount of insulin was analyzed by ELISA method by using insulin antibody. Dissociation constant (K_d_) and binding capacity (n = number of insulin binding molecules/casein) was analyzed from Scatchard plot. From the analysis it was found a curvilinear plot of heterogeneous binding sites population of insulin on casein.

Scatchard plot analysis of the equilibrium binding of insulin to the pure casein produce a typical curvature plot similar to that of insulin receptors binding (Fig. 3), demonstrating that the interaction between the insulin and the casein followed a negative cooperativity as described in method between the casein and insulin binding sites in the casein. Analysis of scatchard plot binding of insulin to casein showed the heterogeneous binding sites of insulin in the casein molecule. A high affinity binding site (K_d1_ = 12.36 pM) with low capacity insulin binding site (n_1_ = 6.02 × 10^19^molecules/casein) and a low affinity (K_d2_ = 43.96 pM) with high capacity binding site (n_2_ = 11.08 × 10^19^molecules/casein) were found.

## Discussion

It was found that the incubation of insulin with the milk at 23°C at least for two and half hours was essential before the milk-insulin was treated with 0.6M acetic acid to precipitate proteins when the insulin milk mixture was treated with acetic acid without pre-incubation and the precipited to proteins were resuspended in the same milk preparation and fed to the alloxan treated mice no hypoglycemic effect on the insulin preparation as described above could be found. In control experiments the treatment of the milk with acetic acid alone had no hypoglycemic effect either when insulin was not added to the milk.

Furthermore it was found that the use of lemon juice (20 μL) instead of acetic acid in the milk and insulin mixture to precipitate the protein, produce hypoglycemic effect similar to the case of acetic acid.

That milk could have protective effect on the tryptic digestion in the digestive tract has been suggested before^13^,^14^. It has been suggested that mother’s milk protects insulin against tryptic digestion in new born babies^15^,^16^ our results as described concluded that casein due to its high affinity binding to insulin was able to co-precipitated with casein by acetic acid in the digestive tract of alloxan treated diabetic mice and the casein bound hormone protected from the tryptic digestion in the digestive tract of the animal. One of the most important steps of our experiment was the incubation of milk with insulin and precipitation by acetic acid. Protein must be precipitated as during the precipitation of protein, H-bonds in the casein molecules were collapsed and bound to insulin with high affinity (≈13pM) and as such serine protease could not get access to the insulin molecule for its enzymic effect.

Intestinal loop experiment also demonstrated that our oral insulin came out from the mice gut to the circulation due to the thermodynamically favored system which was based on the mechanism of Gibbs free energy (∆G) of the reaction that was energy independent facilitated diffusion mediated by transport protein embedded in the cellular membrane.

The result presented in Table 1 clearly demonstrated that milk-insulin was evenly distributed in various arteries of the experimental mice. Insulin was thoroughly and uniformly distributed in all over the system of the animal. We preferred this method rather than tagged insulin by using I-125 insulin, because in our previous experience, I-125 insulin produced high background noise. In that sense ELISA assay of insulin is more appropriate and produce very low background noise.

Instead of painless modern injection, the inconvenience and uneasiness of insulin injection cannot be overlooked. However, as the diabetic person needs to repeat insulin injection continuously in life time and as such insulin injection might cause other problems specially bruising, soreness, infection, redness, irritation occurs at the site of injection. If it does not administer correctly, muscle cramp may cause. Lipohypertrophy, is one of the common side effect at the injection site, where overgrowth of fat cells often found that makes the skin look lumpy. Sometimes it can also look as scared tissue. We are sometime reluctant to change the site because we feel less painful in that area as hypertrophy can numb the area. Sometime injection may be more painful in that area not only, but abnormal cell growth can also limit the insulin absorption. In that sense our oral insulin could be safer and alternative to the insulin injection. From the above experiment it was found that our oral insulin is basal insulin which demonstrated from its duration of action.

As described above the oral insulin preparation contained only common food items and injectable recombinant insulin preparation available commercially. And, as such, the oral insulin preparation as described might be useful for the control of hyperglycemia in diabetes mellituses instead of injection of the hormone, uncomfortable and century old procedure both for the young and old victims with the condition particularly for the economically disadvantaged peoples in the poorer countries all over the world.

## Additional Information

**How to cite this article**: Bank, S. *et al*. The control of hyperglycemia by a novel trypsin resistant oral insulin preparation in alloxan induced type I diabetic mice. *Sci. Rep.* **6**, 26789; doi: 10.1038/srep26789 (2016).

## Supplementary Material

Supplementary Information

## Figures and Tables

**Table 1:** Distribution of insulin in different arteries of the diabetic mice after feeding the milk-insulin to the mice.

Blood samples from artery and vein from different parts of the body	Distribution of insulin (μUnits/ml) at different time intervals		
	0 h	3 h	7 h
Tail vein	3 ± 1.1	13 ± 1.4	18 ± 1.5
Left & right superior vena cava	2.8 ± 1.2	14.2 ± 1.6	18 ± 2.2
Hepatic vein	4 ± 1.7	13.8 ± 1.9	17.1 ± 2.0
Femoral vein	3.5 ± 1.3	14 ± 1.1	17.3 ± 1.4

Milk-insulin preparation solution was prepared as described in Method and Material section. The solution was fed to the diabetic mice as described in method and blood was taken from different parts of the body at different time intervals. The amount of insulin in the blood samples were determined by ELISA using insulin antibody.
