# Supplementary material for: The control of hyperglycemia by a novel trypsin resistant oral insulin preparation in alloxan induced type I diabetic mice
Source: Sci Rep. 2016 May 26;6:26789. doi: 10.1038/srep26789 (PMC4881006; doi:10.1038/srep26789)
Supplement: Supplementary Information [file srep26789-s1.pdf]

Title: The control of hyperglycemia by a novel trypsin resistant oral insulin preparation in alloxan induced type I diabetic mice.

Authors: Sarbashri Bank[1,2], Arjun Ghosh[1], Suman Bhattacharya[1], Smarajit Maiti[2], Gausal A Khan[3], Asru K Sinha[1§]

Affiliation: [1]Sinha Institute of Medical Science & Technology
288-Kendua main road, Baishnabghata, Garia, Kolkata-700084, India

[2]Cell & Molecular Therapeutic Lab, Dept. of Biochemistry, Vidyasagar University[2], Midnapur-721102

[3]Department of Physiology, Defence Institute of Physiology and Allied Sciences[3], Timarpur, Delhi, India.

[§]Corresponding author:

Prof. Asru K Sinha, D.Sc.

Sinha Institute of Medical Science & Technology
288-Kendua main road, Baishnabghata, Garia, Kolkata-700084
Phone-+919038975945, India
Fax: 033-28371048
Email: asruksinha@yahoo.com

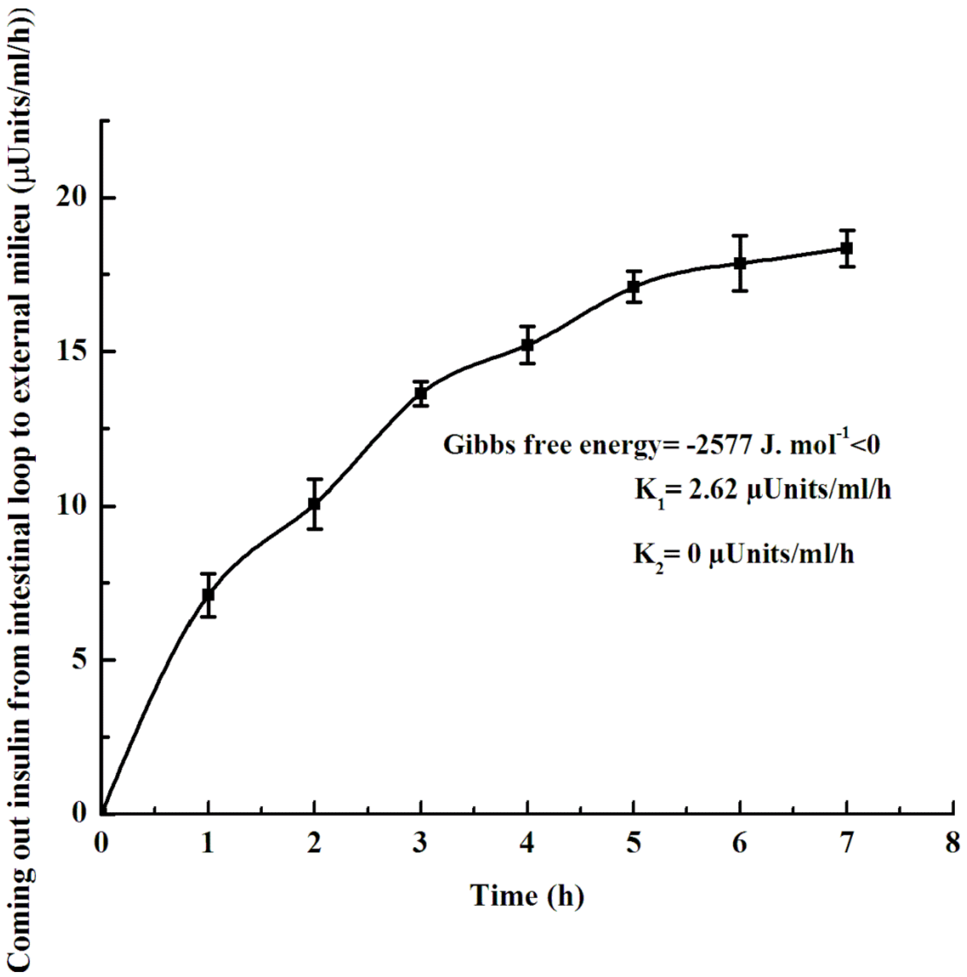

Supplementary Fig. S1: Coming out of milk-insulin preparation from the intestinal loop to the external milieu was a facilitated diffusion

The amount of insulin came out from intestinal loop to the external milieu was analyzed by enzyme linked immunosorbant assay using insulin antibody. From the assay, it was found that the rate of coming out of insulin (K_1) from the intestinal loop was continuously increased in each hour (2.62 µUnits of insulin/ml/h) and the backward movement K_2 was undectable (0 µUnits of insulin/ml/h) was found like a hyperbolic curve which demonstrated that the phenomenon was

not an simple diffusion but energy independent thermodynamically favored (ΔG= -2577 J. mol^{-1}, 0>G indicating a spontaneous reaction) facilitated diffusion transportation.
